# Cost talk: protocol for a stepped-wedge cluster randomized trial of an intervention helping patients and urologic surgeons discuss costs of care for slow-growing prostate cancer during shared decision-making

**DOI:** 10.1186/s13063-021-05369-4

**Published:** 2021-06-29

**Authors:** Mary C. Politi, Rachel C. Forcino, Katelyn Parrish, Marie-Anne Durand, A. James O’Malley, Glyn Elwyn

**Affiliations:** 1 Division of Public Health Sciences, Department of Surgery, Washington University School of Medicine, 660 S. Euclid Ave., Campus Box 8100, St. Louis, MO 63110 USA; 2 The Dartmouth Institute for Health Policy and Clinical Practice, Geisel School of Medicine at Dartmouth, Dartmouth College, Lebanon, NH USA; 3 Université Toulouse III Paul Sabatier, Toulouse, France; 4 Department of Biomedical Data Science, Geisel School of Medicine at Dartmouth, Dartmouth College, Lebanon, NH USA

**Keywords:** Stepped-wedge cluster randomized trial, Costs of care, Prostate cancer, Shared decision-making, Clinical communication, Financial toxicity, Cost conversations

## Abstract

**Background:**

Costs of care are important to patients making cancer treatment decisions, but clinicians often do not feel prepared to discuss treatment costs. We aim to (1) assess the impact of a conversation-based decision aid (Option Grid) containing cost information about slow-growing prostate cancer management options, combined with urologic surgeon training, on the frequency and quality of patient-urologic surgeon cost conversations, and (2) examine the impact of the decision aid and surgeon training on decision quality.

**Methods:**

We will conduct a stepped-wedge cluster randomized trial in outpatient urology practices affiliated with a large academic medical center in the USA. We will randomize five urologic surgeons to four intervention sequences and enroll their patients with a first-time diagnosis of slow-growing prostate cancer independently at each period. Primary outcomes include frequency of cost conversations, initiator of cost conversations, and whether or not a referral is made to address costs. These outcomes will be collected by patient report (post-visit survey) and by observation (audio-recorded clinic visits) with consent. Other outcomes include the following: patient-reported decisional conflict post-visit and at 3-month follow-up, decision regret at 3-month follow-up, shared decision-making post-visit, communication post-visit, and financial toxicity post-visit and at 3-month follow-up; clinician-reported attitudes about shared decision-making before and after the study, and feasibility of sustained intervention use. We will use hierarchical regression analysis to assess patient-level outcomes, including urologic surgeon as a random effect to account for clustering of patient participants.

**Discussion:**

This study evaluates a two-part intervention to improve cost discussions between urologic surgeons and patients when deciding how to manage slow-growing prostate cancer. Establishing the effectiveness of the strategy under study will allow for its replication in other clinical decision contexts.

**Trial registration:**

ClinicalTrials.govNCT04397016. Registered on 21 May 2020

**Supplementary Information:**

The online version contains supplementary material available at 10.1186/s13063-021-05369-4.

## Background

Financial toxicity refers to patients’ distress resulting from the costs of cancer care [1]. Direct and indirect treatment costs contribute to financial toxicity among people with cancer [2, 3]. Financial toxicity is associated with both poorer clinical outcomes [1, 4, 5] and poorer quality of life in cancer survivors [6, 7] and has an impact for many years after diagnosis [8]. Many patients with cancer report financial toxicity [8]. Its prevalence has made addressing the costs of care a priority for patients [9] and healthcare organizations [10, 11].

Patient-clinician discussions about treatment costs can help lower costs of care and reduce financial toxicity [1]. Patients welcome opportunities to discuss treatment costs with their clinicians [9, 12], but few patients and clinicians engage in cost conversations in routine practice [13–15]. As a result, many patients with prostate cancer pay more for their treatment than they expected [16]. While clinicians acknowledge the importance of addressing financial toxicity [17], they feel underprepared to initiate or lead cost conversations [13].

Preparing clinicians for cost discussions is an important component of reducing patients’ financial toxicity. After addressing costs during treatment discussions, clinicians can seek support from other professionals, such as social workers or financial navigators, to provide details about personal costs based on insurance and health needs [18]. Referring the patient for additional advice is a key outcome of initiating cost conversations.

To address the gap between guidelines, needs, and practice, in this study, we will train urologic surgeons in the use of a conversation-based decision aid containing cost-related resources to discuss costs when engaging in shared decision-making (SDM) for slow-growing (also called low-risk) prostate cancer. We will examine the resulting frequency and content of cost discussions between urologic surgeons and patients as they discuss this preference-sensitive treatment decision in the context of SDM. Results can provide insights into how to address cost conversations during SDM about cancer care more broadly.

## Objectives

### Aim 1

We aim to assess the impact of a conversation-based decision aid (Option Grid) containing cost information about slow-growing prostate cancer management options, combined with a brief training session for the urologic surgeon, on the frequency and quality of patient-urologic surgeon cost conversations. We will measure frequency of cost conversations, initiator of cost conversations, and whether or not a referral is made to address costs. We hypothesize that:

- 1.1: Urologic surgeons assigned to training and use of the decision aid will engage in more frequent cost conversations than urologic surgeons in usual care.
- 1.2: Urologic surgeons assigned to training and use of the decision aid will be more likely than patients to initiate cost conversations. Patients will be more likely than urologic surgeons to initiate cost conversations in usual care.
- 1.3: Urologic surgeons assigned to training and use of the decision aid will be more likely to make a referral to address specific cost details than urologic surgeons in usual care.
- 1.4 (exploratory): Patients of urologic surgeons assigned to training and use of the decision aid will have lower financial toxicity at three-month follow-up than patients of urologic surgeons in usual care.

### Aim 2

We aim to examine the impact of the conversation-based decision aid and surgeon training on decision quality, including measures of decisional conflict, decision regret, and shared decision-making. We hypothesize that:

- 2.1: Patients of urologic surgeons assigned to training and use of the decision aid will report less decisional conflict, less decisional regret at three-month follow-up, and more SDM than patients in usual care.

## Methods

### Design

**Fig. 1:**
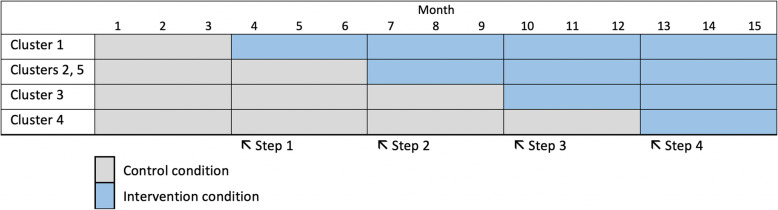
Study overview

This study uses a stepped-wedge cluster-randomized trial design (see Fig. 1) [19]. There are four sequences, with at least one cluster (urologic surgeon) randomized to each sequence. The fifth urologic surgeon will be assigned to the second sequence in order to balance patient accrual to the intervention and control arms. There are five periods, each lasting 3 months, for a total study duration of approximately 15 months. Independent eligible patients will be enrolled at each period within a cluster (urologic surgeon).

We clustered intervention assignment at the urologic surgeon level because the intervention includes urologic surgeon training and is designed to impact clinical communication patterns, which are reflected in the study outcomes. This clustering approach will minimize the likelihood of contamination between study arms that could occur with patient-level randomization.

The staggered assignment of participating urologic surgeons to the intervention arm has several advantages. The stepped-wedge design allows more surgeons to undergo training in use of the decision aid intervention than would a parallel trial design with a usual care control arm. This is an advantage in the context of strong interest in learning about decision aids in the participating study setting paired with evidence that decision aids improve patient-centered care [20]. Additionally, the staggered timing of surgeon training will facilitate its scheduling within busy clinician timetables. This improves overall study feasibility while ensuring a consistent intervention dose for all participating surgeons.

### Setting

We will conduct this study in outpatient practices affiliated with a large academic medical center. These participating practices are located in a single metropolitan area in the Midwest region of the USA. Decision aids were not routinely used in the participating practices prior to study participation.

### Participants

#### Included urologic surgeons

We will recruit five urologic surgeons who routinely discuss management options for slow-growing prostate cancer with patients.

#### Included patients

We will recruit up to 200 patient participants based on projected volume of eligible patients during each period. Eligible patients include adults visiting a participating urologic surgeon to discuss management options for a first-time diagnosis of slow-growing prostate cancer. Slow-growing prostate cancer diagnoses will initially be defined by a Gleason score of 6 or 7 (3 + 4) and/or prostate-specific antigen (PSA) level less than 10 ng/ml, and/or as confirmed by a surgeon’s referral prior to patient recruitment. Eligible patient visits may occur in-person or by telehealth. We will exclude patients who cannot give informed consent due to cognitive or emotional barriers, and those who are discussing recurrent or ongoing prostate cancer management. Patients seen in these practices are from urban, suburban, and rural settings (approximately 14% from rural settings and approximately 25% from a medically underserved area). Patients in these practices are racially diverse, with approximately 20% identifying as Black or African American, 3% identifying as Hispanic of any race, and 4% identifying as two or more races.

### Intervention

This study tests a two-part intervention: (1) use of an Option Grid conversation-based decision aid by urologic surgeons and patients during their first consultation about managing slow-growing prostate cancer and (2) brief urologic surgeon training in shared decision-making, patient-facing financial resources, and best practices for conversation-based decision aid use.

#### Option Grid conversation-based decision aid with cost information

The Option Grid decision aid is a table with side-by-side comparisons of management options for slow-growing prostate cancer (specified in Table 1), organized as responses to patients’ frequently asked questions [21]. Written at a sixth grade reading level, the grid was developed, tested, and validated using a systematic process including evidence summarization and stakeholder input [21–23]. This type of decision aid is not designed to be comprehensive, but to supplement clinicians’ explanations [24]. For this study, we have added two types of cost information to the Option Grid: (1) general descriptions of relative costs [25] for each treatment/management option (see Tables 1 and 2) a list of general and local resources for navigating cancer care costs. Participating urologic surgeons will deliver the Option Grid intervention at the individual patient level.

**Table 1:** Relative cost information added to the Slow-Growing Prostate Cancer Option Grid

Option	Included relative cost information
Monitor with tests	Costs include testing at least once a year. Check your insurance for your exact costs. $-$$
Prostatectomy	Costs include surgery and hospital stay, up to 3 days. Check your insurance for your exact costs. $$$
Radiation (external beam radiation therapy or brachytherapy)	Check your insurance for your exact costs. *External beam radiation*: $$$ *Brachytherapy:* $$-$$$
**Included cost resources**	
Health insurance can help cover some of your care costs. Whatever type of insurance you have, you want to get the most from your plan. You should call your plan to find out things like: • Does my plan cover the care I need? • Are the hospital and doctors who will be treating me part of my plan’s network? • Does my plan cover the facility fee? If so, how much of the fee is covered? • Do I need to be pre-approved by the insurance company before getting this care? • How much of my deductible have I met? How much of my deductible is left before I meet it? • What will be the copay or coinsurance for the treatment I need? In many cancer centers, social workers or navigators can help patients find information about costs or apply for help paying your bills. They can also help if you need a way to get to and from treatment, short-term housing, or have problems with your job because of your care. For more information or to reach a social worker at [cancer center name], you can call [phone number]. This website also has resources for patients at [cancer center name], including how to apply for more help paying your bills. [Web address for cancer center’s patient support services]

**Table 2:** Study data summary

Domain (measure)	Source	Timing
**Primary outcomes**		
Frequency of cost conversations (adapted Politi [14]/Hunter [26] checklist)	Patient report; observation	Immediately post-visit
Initiator of cost conversations (adapted Politi [14]/Hunter [26] checklist)	Patient report; observation	Immediately post-visit
Referral to address costs (adapted Politi [14]/Hunter [26] checklist)	Patient report; observation	Immediately post-visit
**Secondary outcomes**		
Shared decision-making (collaboRATE [27])	Patient report	Immediately post-visit
Effort made to compare costs of treatment options	Patient report	Immediately post-visit
Clinical communication (CAHPS communication composite [28])	Patient report	Immediately post-visit
Decisional conflict (SURE [29])	Patient report	Immediately post-visit, 3 months post-visit
Decision regret (Decision Regret Scale [30])	Patient report	3 months post-visit
**Exploratory outcomes**		
Financial toxicity (COST [31, 32])	Patient report	Immediately post visit; 3 months post-visit
**Implementation and acceptability outcomes**		
Attitudes toward SDM (Continuing Professional Development Reaction Scale [33])	Clinician report	Beginning of study period; end of study period
Feasibility of sustained cost conversations and Option Grid use	Clinician report	End of study period
Acceptability of cost conversations and Option Grid use (adapted Ottawa Acceptability Scale [34])	Clinician report	End of study period
**Other data**		
Treatment choice	Patient report	Immediately post-visit
Treatment received	Electronic health record	3 months post-visit
Health literacy (Single-Item Literacy Screener [35])	Patient report	Immediately post-visit
Patients’ other (comorbid) health conditions	Electronic health record	Immediately post-visit
Patients’ demographic characteristics	Patient report	Immediately post-visit

#### Option Grid training

At the start of the study, urologic surgeons will be trained in the study protocol. At the start of their active arm assignment, each surgeon will complete a survey about their attitudes relating to SDM and will be trained in use of the Option Grid, including discussing costs with patients. Option Grid training will (1) present a model of SDM [36] and best practices for use of the Option Grid; (2) share details about incorporating cost discussions into SDM; (3) provide each surgeon with a list of cost-related resources and referrals for patients, including ways to lower out-of-pocket costs; and (4) include a mock patient simulation to allow the urologic surgeon to practice using the intervention. The study’s principal investigator (MCP) and co-principal investigator (GE) will deliver the Option Grid training at the cluster level.

### Outcomes

#### Primary outcomes

We will measure the frequency of cost conversations, initiator of cost conversations, and whether or not a referral is made to address costs. We will collect these primary outcomes by patient report from all participating patients in a questionnaire completed immediately post-visit (or shortly after a telehealth visit).

We will also use an existing observer checklist [14] to assess these outcomes for participating patients who consent to audio-recording their clinic visits. We will use Hunter et al.’s approach to defining a cost discussion: “any mention of the patient’s out-of-pocket expenses or insurance coverage for a past, present, or potential health care service” [26]. For example, any mention of costs or insurance coverage for primary surgery, radiation, imaging, as well as indirect costs of care such as time off work, transportation, recovery time, will be documented and included in our analyses. If direct or indirect costs are mentioned per Hunter’s definition [26], we will indicate that costs were discussed during the consultation. If there are two mentions of cost in one transcript, with a different topic in between, the mentions will be coded as two cost discussions. If costs are discussed at all, the subsequent items (length of time of the cost conversation, number of times cost was discussed, who initiated the cost discussion, content of cost discussions, whether a referral is made to address costs) will then be evaluated. We will document references to the Option Grid to examine whether the Option Grid directly prompted the cost conversation. Because we do not expect surgeons to know specific details about treatment costs, the checklist also includes an item to measure whether a referral is made to address more detail about costs. We will document to whom a referral is made (navigator, social worker, outside resource).

#### Secondary outcomes

##### Patient-reported

Within 24 h after the clinic visit, we will collect the following patient-reported outcomes: the SURE measure of decisional conflict [29], the collaboRATE measure of SDM [27], a single item added to collaboRATE to assess effort to compare costs of treatment, the CAHPS measure of communication quality [28], and the COST measure of financial toxicity [31, 32]. Financial toxicity will be an exploratory outcome.

At 3-month follow-up, we will collect the following patient-reported outcomes: the SURE measure of decisional conflict [29], the decision regret scale [30], and the COST measure of financial toxicity [31, 32]. Financial toxicity will be an exploratory outcome.

##### Clinician-reported

Participating urologic surgeons will complete a 12-item measure of SDM perceptions [33] at the start of their arm assignment and again at the end of the study period. Each study urologic surgeon will also complete an adaptation of the Ottawa Acceptability Scale [34] and survey items about feasibility of routine Option Grid use at the end of the study period.

Table 2 summarizes all study measurement, including outcomes and planned covariates.

### Randomization

We will randomly allocate clusters (urologic surgeons) to the intervention sequences with a simple randomization approach (i.e., no blocking, stratification, or other constraints) using R statistical software. Randomization will take place after urologic surgeon recruitment is complete; both participating urologic surgeons and the study team are therefore blinded to allocation until after urologic surgeon recruitment. The study coordinator (KP) will enroll clusters (urologic surgeons) to the study. The study statistician (AJO), blinded to the identity of each cluster, will generate the randomization schedule and assign cluster IDs to sequences.

Eligible patients will be recruited continuously throughout the study period; the study coordinator (KP) will identify eligible patients and seek their informed consent to participate during both control and intervention periods. In order to manage logistics of implementing the intervention and recruiting patient participants, the principal investigators (MCP, GE), study coordinator (KP), and participating urologic surgeons will learn the allocated sequence assignments immediately post-randomization. Patients will not be informed of their study arm allocation during the recruitment process, but will not be blinded as they will observe whether or not they receive the Option Grid intervention at the time of their visit. Other members of the study team, including those analyzing results, will remain blinded to cluster sequence allocation throughout the study.

### Procedures

**Fig. 2:**
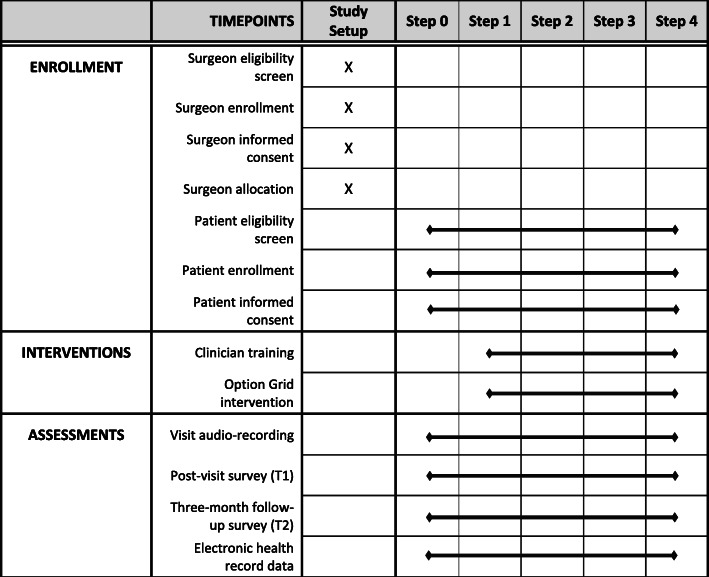
Schedule of enrollment, interventions, and assessments

Figure 2 summarizes study enrollment, interventions, and assessments.

#### Stakeholder advisory group

A stakeholder advisory group comprising patients, clinicians, and prostate cancer advocates has informed our study design, outcomes, and procedures. This group will meet every 1 to 2 months throughout the study period to co-design study materials, monitor study progress, and troubleshoot problems where necessary. We compensate these stakeholder partners at a rate that reflects their substantial expertise and commitment.

#### Recruitment

##### Clinician recruitment

Urologic surgeons who wish to participate will register their interest with the urology division chief, who will share their contact details with the principal investigator (MCP) to facilitate enrollment. In the weeks prior to the start of the study period, the study coordinator (KP) will seek written informed consent from interested and eligible urologic surgeons by phone and/or email.

##### Patient recruitment

We will recruit patient participants from July 2020 through September 2021. The study coordinator will identify potentially eligible patients with upcoming clinic visits using clinic schedules, electronic health records, contact with clinicians, and contact with clinic coordinators, then confirm eligibility with the patient’s urologic surgeon. The study coordinator will retrieve phone numbers for eligible patients from the electronic health record. To assess interest in participating, the study coordinator will contact each eligible patient by phone before their scheduled visit, but after the care team has communicated a prostate cancer diagnosis to the patient. To reduce risk of selection bias due to unblinded treatment allocation, the study coordinator will approach all eligible patients, defined as men with prostate cancer who have a Gleason 6 or 7 and/or PSA less than or equal to 10 ng/ml, or as referred by a surgeon whose clinical expertise confirms that the individual is eligible for the options in the Option Grid intervention. If the eligible patient is interested in participating, the study coordinator will document informed consent over the phone, electronically as a waiver of written consent for those who agree to be emailed, or in-person when the patient arrives for the clinic visit.

#### Data collection

##### Observational data

For participating patients who agree during the study consent process to audio-recording of their in-person clinic visits, the study coordinator will start a digital recorder when the patient enters the exam room and return to collect the recorder at the end of the clinic visit. Where participating patients agree to audio-recording of their virtual visits, audio-recordings will be captured by the urologic surgeon through Zoom or other HIPAA-compliant telemedicine software and subsequently transferred securely to the study coordinator.

Audio-recordings will be transcribed verbatim for analysis. Two members of the study team will independently review each transcript and apply the observer checklist [14, 26] to identify frequency and initiator of cost conversations and frequency of referrals to address costs.

At the time of patient enrollment, study staff will review the electronic health record to determine whether or not the participant has been diagnosed with each of the 20 most common and costly health conditions, as described in the Medical Panel Expenditure Survey [37–39], and record those comorbidities in the study’s Research Electronic Data Capture database (REDCap). At 3-month follow-up, study staff will collect the patient’s received treatment (if any) from the electronic health record and record that information in REDCap.

##### Patient-reported data

We will administer post-visit patient questionnaires at two timepoints: immediately after the clinic visit and 3 months after the clinic visit. At both timepoints, study staff will collect patient-reported data by emailed link to an online survey form hosted in REDCap or by standardized telephone interview, depending on participant preference. All questionnaire data will be stored in REDCap and response modality will be recorded. Study staff will make up to five attempts to contact patient participants for response to each survey.

After completion of the first post-visit questionnaire, study staff will mail patient participants a $15 gift card. After completion of the second questionnaire at 3-month follow-up, study staff will mail patient participants a $5 gift card.

##### Clinician-reported data

For each set of clinician-reported outcomes, the study coordinator will email a link to an online survey hosted in REDCap to each participating urologic surgeon. The study team will send email reminders as needed until responses are collected from each participating urologic surgeon. Upon completion of all study procedures, the study team will provide a $50 gift card to each participating urologic surgeon at the end of the study period.

#### Intervention implementation

##### Option Grid training

At the start of the study period, while all urologic surgeons are allocated to the usual care control condition, study staff will schedule an Option Grid training session with each urologic surgeon to be held at the beginning of his or her designated intervention period. Training sessions will be led by the principal investigators (MCP and GE) and held in-person and/or by Zoom videoconference.

##### Option Grid delivery

Participating urologic surgeons will deliver the Option Grid to all their patients who face a management decision for slow-growing prostate cancer. Some urologic surgeons will direct patients to access the Option Grid through a secure web link prior to their visits, while others will send the Option Grid to patients by postal mail or through the online patient portal. At the time of patient enrollment, the study coordinator will confirm that each participating patient has received the Option Grid from their care team. If a participating patient has not received the Option Grid, study staff will provide it by email or postal mail prior to the patient’s visit.

### Sample size

We assume given the study timeline and projected patient volumes that it is feasible to recruit up to 50 patients from each of the five participating urologic surgeons, to target a total of 200 patient participants. Due to the novelty of our shared decision-making intervention intended to promote cost conversations in cancer care, limited data were available to inform the assumed ICC [14]. Based on the unmeasured clinician-level variability observed in other studies using similar interventions to study different outcomes, we conservatively assumed a relatively large ICC value of 0.05. Under these assumptions, and assuming an intraclass correlation coefficient of 0.05 for our primary outcome, the design effect of the stepped-wedge design equals 3.12, making the effective sample 32 per intervention group; this is the equivalent sample-size under a design in which there is no clustering of patients within surgeons [40].

In relation to Aim 1 and our primary research questions, prior research demonstrates that surgeons who used an encounter decision aid about early-stage breast cancer surgery with comparative cost information were far more likely to engage in cost conversations compared to those using a decision aid without cost information or those in usual care (66.7% versus 33.3%) [14]. Surgeons in the cost information decision aid group were also more likely to initiate these conversations compared to those in the non-cost information decision aid group (86.4% versus 34.1%) [14]. We estimated power directly for a stepped-wedge design. A requirement for performing this calculation is supplying the between-cluster variance. We estimated this quantity by solving for its value given the specified ICC of 0.05 and the within-cluster variance of a Bernoulli (binary-valued) random variable, whose mean equals the average value of the assumed probabilities of the outcomes for the two comparison groups (0.5, the most conservative value we could assume). The resulting power for a two-sided test was estimated to be 0.804. A projected sample size of 200 is therefore sufficient to answer our primary research questions.

Our two-step approach to the included sample size calculation involved mathematical approximations, which may reduce its reliability. While the alternate method of calculating power using a simulation model may have improved the reliability of our sample size calculation in some ways, that approach would require additional assumptions about the effects of time and other covariates on the basis of little available empirical evidence, which may also reduce overall reliability. Therefore, we favor the simpler calculation.

Because of the low-risk nature of the intervention with regard to likelihood and magnitude of potential harms, we do not plan to conduct interim analyses and have not established stopping guidelines.

### Analysis

We will perform unadjusted bivariate comparisons of outcomes and predictors across intervention groups using t tests for continuous variables and chi-square tests for categorical variables. We will also conduct analysis adjusting for patient demographic characteristics, patient clinical characteristics, and the time period when the patient began follow-up. We will use logistic regression to model the Aim 1 primary outcome of whether or not a cost conversation occurred. In Aim 2, we will use linear regression to analyze outcomes with multi-level scales (e.g., decision regret scale [30]) and logistic regression to analyze binary outcome measures (e.g., collaboRATE [41]). To account for clustering of patient participants by urologic surgeon, we will estimate hierarchical models with urologic surgeon random effects. Let *Y*_*ijt*_ denote an outcome measured on the *i*th patient of the *j*th surgeon at time *t* and *OG*_*jt*_ indicate whether surgeon *j* has transitioned from usual care to the Option Grid (OG) by time period *t*. To lessen the reliance on the random assignment of surgeons to step times to balance any changes across time between the intervention groups, we adjust for the time period when the patient began follow-up, patient demographic and patient clinical characteristics. Adjustment for time period will be enabled by including indicator variables for time periods 2 through 5 (making time period 1 the excluded category) with effects denoted by {*λ*_*t*_}=_*t* = 2 : 5_ while all other patient-level variables will be included in a vector of covariates, denoted *X*_*ijt*_. Therefore, the model for the Aim 1 analysis and those outcomes modeled as binary random variables in Aim 2 has the form

1 $$ logit\left(\Pr \left({Y}_{ijt}=1\left|{\theta}_j\right.\right)\right)={\beta}_0+{\beta}_1{OG}_{jt}+{\beta}_2{X}_{ijt}+{\lambda}_t+{\theta}_j $$

while the model for those outcomes modeled as continuous variables in Aim 2 has the form

2 $$ {Y}_{ijt}={\beta}_0+{\beta}_1{OG}_{jt}+{\beta}_2{X}_{ijt}+{\lambda}_t+{\theta}_j+{\varepsilon}_{ijt} $$

where for both models *θ*_*j*_ is a random effect specific to surgeon *j* and for the linear regression model ∈_*ijt*_ is an idiosyncratic error term. The surgeon random effects and the error terms are assumed to be independent normally distributed random variables with unknown variance. Because 5 surgeons is a small number of units in which to estimate a random effect, as a sensitivity analysis we will estimate the generalized estimating equation alternative to these mixed effect models. We will favor the approach that yields the more conservative results.

We will check for outliers and missing data. If there is extensive missing data, we will use one of the following remedies: (1) inverse-probability weighting if there is a single form of missing data (e.g., only the outcome is missing) or (2) multiple imputation if missingness occurs sporadically across the outcomes and predictors. Analyses will follow the intention to treat principle. Where required to make the skewed distribution of a variable more symmetric, we will employ variable transformation.

## Discussion

This study evaluates a strategy to improve the frequency and content of cost conversations between urologic surgeons and patients in preference sensitive decisions about managing slow-growing prostate cancer. This research fills an important gap, as patients and professional organizations alike prioritize cost conversations [9–12] but urologic surgeons can feel ill-equipped to initiate them [17]. Establishing the effectiveness of the strategy under study is an important first step to replicating and/or adapting this approach to other clinical decision contexts.

### Practical issues in performing the study

COVID-19 has impacted clinical research worldwide, with many non-urgent healthcare visits canceled, postponed, or moved to a telehealth format in early-mid 2020. These changes allowed for a lower risk of viral spread within healthcare facilities while directing healthcare resources to COVID-19 treatment. As COVID-19 infection counts stabilize, eligible patient volumes are beginning to return to pre-pandemic levels at the participating study setting. However, a second wave of the virus during the study period could limit our ability to recruit patient participants and meet accrual targets according to our planned timeline. Our inclusion of telehealth visits in this study has the potential to mitigate a slowdown of in-person patient recruitment; however, it can complicate the previously planned in-person procedures. We will document the number of patients seen via telehealth and in-person, and we will explore the potential impact of telehealth on outcomes.

The 2-year study timeline could limit our ability to assess outcomes at long term follow-up. As most active surveillance costs do not accrue immediately, financial toxicity data reported by patients on active surveillance at 3-month follow-up may not be comprehensive; we have therefore included financial toxicity as an exploratory outcome in this study.

## Trial status

The trial is registered at clinicaltrials.gov (NCT04397016): protocol version 1, 21 May 2020. Recruitment began on 24 June 2020 and is expected to end by 30 September 2021.

## Supplementary Information

**Additional file 1.**

**Additional file 2.**

## Data Availability

Data and materials will not be made publicly available.
