# Supplementary material for: Cost talk: protocol for a stepped-wedge cluster randomized trial of an intervention helping patients and urologic surgeons discuss costs of care for slow-growing prostate cancer during shared decision-making
Source: Trials. 2021 Jun 29;22:422. doi: 10.1186/s13063-021-05369-4 (PMC8240421; doi:10.1186/s13063-021-05369-4)
Supplement: Supplementary file 1 — Additional file 1. [file 13063_2021_5369_MOESM1_ESM.pdf]

INFORMED CONSENT DOCUMENT

Project Title: Cost talk: a randomized stepped wedge trial of interventions helping patients discuss cancer care costs with clinicians during shared decision making

Principal Investigator: Mary Politi, PhD at [phone number]

Research Team Contact: Katie Parrish, MPH at [phone number]

This consent form describes the research study and helps you decide if you want to participate. It provides important information about what you will be asked to do during the study, about the risks and benefits of the study, and about your rights and responsibilities as a research participant.

- You should read and understand the information in this document including the procedures, risks and potential benefits.
- If you have questions about anything in this form, you should ask the research team for more information before you agree to participate.
- You may also wish to talk to your family or friends about your participation in this study.
- Do not agree to participate in this study unless the research team has answered your questions and you decide that you want to be part of this study.

Key Information

This is a research study conducted by Mary Politi, PhD. The goal of this study is to understand how a patient decision aid for early-stage prostate cancer treatment affects care cost discussions. You should think about the information in this consent and discuss it with the research team. You should understand why you might want to join the study, or why you might not want to join. You may choose to join or not join.

Joining the study is completely up to you. If you agree to join the study, you will be asked to allow your clinic visits with eligible patient participants to be audio-recorded and to complete training on the study protocol, use of the Option Grid patient decision aid, and costs of care/financial resources. The main risk to you if you join the study is that confidential information about you may be accidentally disclosed.

We don't expect this study to benefit you directly, but it will help us understand how to better support patients as they talk about care costs and find resources to help with those costs. There is no cost to you. You will receive a one-time payment in the form of a $50 gift card at the end of the study. All of this information will be explained and is listed in more detail in this consent. The research team will provide you with a copy of this consent.

WHAT IS THE PURPOSE OF THIS STUDY?

This is a research study. We invite you to participate in this research study because you counsel and treat patients with slow-growing prostate cancer.

The purpose of this study is to understand how a patient decision aid for prostate cancer treatment affects care discussions.

WHAT WILL HAPPEN DURING THIS STUDY?

1) You will be asked to complete a 12-item survey.
2) Research staff will start an audio-recorder when a participating patient enters the exam room, then come back at the end of the appointment to turn off the recorder.
3) The Principal Investigators and study staff will conduct Option Grid training sessions, either in-person or by videoconference. Once trained, you will use the Option Grid during your visits with participating patients.
4) At the end of the study, you will be asked to complete the same 12 item survey.
5) Research staff will give you a $50 gift card at the end of the study to thank you for your time.

If at any point in the study you no longer wish to participate, you can stop the recording. You can send us an email or call us by phone to let us know you no longer wish to participate. You will not be penalized or lose any benefits.

If you need more time to think about your participation, we will follow-up with you with a phone call and/or email in two weeks. We will reach out three times before removing you from the study.

Will you save my research information to use in future research studies?

Your private information will NOT be used for future research studies or shared with other researchers for their studies, even if we remove identifiers

Audio/Video Recording or Photographs

One aspect of this study involves making audio recordings of you. This is done to learn from the conversations you are having with your patients to help patients in the future have conversations with their clinicians about cancer care. Only the study team has access to the recordings, which will be destroyed when the study is over. The audio recording is optional and you can still be in the study without being recorded.

I give you permission to make an audio recording of me during this study.

_____ Yes **_____ No**
Initials **Initials**

HOW MANY PEOPLE WILL PARTICIPATE?

Approximately 200 people will take part in this study conducted by investigators at Washington University.

HOW LONG WILL I BE IN THIS STUDY?

If you agree to take part in this study, your involvement will last for the duration of your clinic visits with eligible patient participants.

WHAT ARE THE RISKS OF THIS STUDY?

You may experience one or more of the risks indicated below from being in this study. In addition to these, there may be other unknown risks, or risks that we did not anticipate, associated with being in this study.

One risk of participating in this study is that confidential information about you may be accidentally disclosed. We will use our best efforts to keep the information about you secure. Please see the section in this consent form titled *"How will you keep my information confidential?"* for more information.

WHAT ARE THE BENEFITS OF THIS STUDY?

You will not benefit from being in this study. However, we hope that, in the future, other people might benefit from this study because it may help us understand how to better support patients as they talk about cancer care options.

WILL IT COST ME ANYTHING TO BE IN THIS STUDY?

You will not have any costs for being in this research study.

WILL I BE PAID FOR PARTICIPATING?

You will be paid for being in this research study. You will receive a one-time payment in the form of a $50 gift card at the end of the study.

You will need to provide your social security number (SSN) in order for us to pay you. You may choose to participate without being paid if you do not wish to provide your social security number (SSN) for this purpose. You may also need to provide your address if a gift card will be mailed to you. It will take approximately 2 weeks for the gift card to be delivered. If your social security number is obtained for payment purposes only, it will not be retained for research purposes.

WHO IS FUNDING THIS STUDY?

The Robert Wood Johnson Foundation is funding this research study. This means that the Washington University is receiving payments from the Robert Wood Johnson Foundation to support the activities that are required to conduct the study. No one on the research team will receive a direct payment or increase in salary from the Robert Wood Johnson Foundation for conducting this study.

HOW WILL YOU KEEP MY INFORMATION CONFIDENTIAL?

Other people such as those indicated below may become aware of your participation in this study and may inspect and copy records pertaining to this research. Some of these records could contain information that personally identifies you.

- Government representatives (including the Office for Human Research Protections) to complete federal or state responsibilities
- University representatives to complete University responsibilities
- Washington University's Institutional Review Board (a committee that oversees the conduct of research involving human participants) and Human Research Protection Office. The Institutional

Review Board has reviewed and approved this study.
- Any report or article that we write will not include information that can directly identify you. The journals that publish these reports or articles require that we share your information that was collected for this study with others to make sure the results of this study are correct and help develop new ideas for research. Your information will be shared in a way that cannot directly identify you.

To help protect your confidentiality, we will use an ID code to identify your audio-recordings instead of your name. We will destroy the link between the ID code and your name after the study is over. All data will be stored electronically under password protection in a secured server. Study-related computers will be under firewall protection and will maintain automated virus update mechanisms. Timely notification regarding relevant patches will be provided. Hard copies of data collection forms will be stored in locked cabinets in locked areas to which only authorized personnel will have access. In addition, all study staff annually sign a confidentiality statement attesting to their understanding of, and willingness to abide by, written policies on research ethics and confidentiality. Access to the data entry website will be password protected and restricted to personnel trained to use the system.

Our collaborators at Dartmouth College will be involved in the data analysis once the study is finished, but will only have access to de-identified data that cannot be linked to you.

The Siteman Cancer Center at Washington University School of Medicine and Barnes-Jewish Hospital is supported by funding from the National Cancer Institute (NCI). To meet NCI requirements, your protected health information relating to your participation in this study (including your social security number) will be stored in a secure database at the Siteman Cancer Center. This database and also your health care records may be reviewed by Siteman Cancer Center personnel. All information will be securely and confidentially maintained.

You have the right to share your information or involvement in this study with anyone at any time. You may also give the research team permission to disclose your information to a third party or any other person not connected with the research.

IS BEING IN THIS STUDY VOLUNTARY?

Taking part in this research study is completely voluntary. You may choose not to take part at all. If you decide to be in this study, you may stop participating at any time. Any data that was collected as part of your participation in the study will remain as part of the study records and cannot be removed.

If you decide not to be in this study, or if you stop participating at any time, you won't be penalized or lose any benefits for which you otherwise qualify.

What if I decide to withdraw from the study?

You may withdraw by telling the study team you are no longer interested in participating in the study.

If at any point in the study you no longer wish to participate, you can stop the audio-recording and/or stop answering survey questions. You can send us an email or give us a phone call to let us know you no longer wish to participate. You will not be penalized or lose any benefits for which you otherwise

qualify.

If you do not tell us you no longer wish to participate in the study or need more time to think about your participation, we will follow-up with you with a phone call and/or email in two weeks. We will reach out three times before removing you from the study.

Will I receive new information about the study while participating?

If we obtain any new information during this study that might affect your willingness to continue participating in the study, we'll promptly provide you with that information.

WHAT IF I HAVE QUESTIONS?

We encourage you to ask questions. If you have any questions about the research study itself, please contact: Katie Parrish, MPH at [phone number]. If you feel that you have been harmed in any way by your participation in this study, please contact Mary Politi, PhD at [phone number].

If you have questions, concerns, or complaints about your rights as a research participant please contact the Human Research Protection Office at [phone number], or email [email address]. General information about being a research participant can be found on the Human Research Protection Office web site, http://hrpo.wustl.edu. To offer input about your experiences as a research participant or to speak to someone other than the research staff, call the Human Research Protection Office at the number above.

This consent form is not a contract. It is a written explanation of what will happen during the study if you decide to participate. You are not waiving any legal rights by agreeing to participate in this study.

Your signature indicates that this research study has been explained to you, that your questions have been answered, and that you agree to take part in this study. You will receive a signed copy of this form.

Do not sign this form if today's date is after EXPIRATION DATE: N/A.

____ ______________________________

(Signature of Participant) (Date)

____

(Participant's name – printed)

Statement of Person Who Obtained Consent

The information in this document has been discussed with the participant or, where appropriate, with the participant's legally authorized representative. The participant has indicated that they understand the risks, benefits, and procedures involved with participation in this research study.

____ ______________________________
(Signature of Person who Obtained Consent) (Date)

____
(Name of Person who Obtained Consent - printed)
