# Supplementary material for: Cost talk: protocol for a stepped-wedge cluster randomized trial of an intervention helping patients and urologic surgeons discuss costs of care for slow-growing prostate cancer during shared decision-making
Source: Trials. 2021 Jun 29;22:422. doi: 10.1186/s13063-021-05369-4 (PMC8240421; doi:10.1186/s13063-021-05369-4)
Supplement: Supplementary file 2 — Additional file 2. [file 13063_2021_5369_MOESM2_ESM.pdf]

INFORMED CONSENT DOCUMENT

Project Title: Cost talk: a randomized stepped wedge trial of interventions helping patients discuss cancer care costs with clinicians during shared decision making

Principal Investigator: Mary Politi, PhD at [phone number]

Research Team Contact: Katie Parrish, MPH at [phone number]

This consent form describes the research study and helps you decide if you want to participate. It provides important information about what you will be asked to do during the study, about the risks and benefits of the study, and about your rights and responsibilities as a research participant.

- You should read and understand the information in this document including the procedures, risks and potential benefits.
- If you have questions about anything in this form, you should ask the research team for more information before you agree to participate.
- You may also wish to talk to your family or friends about your participation in this study.
- Do not agree to participate in this study unless the research team has answered your questions and you decide that you want to be part of this study.

Key Information

This is a research study conducted by Mary Politi, PhD. You are being asked to join this research study because you have slow-growing prostate cancer. The goal of this study is to understand whether and how a decision tool about slow-growing prostate cancer affects care cost discussions and patients' care decisions. You should think about the information in this consent and discuss it with the research team. You should understand why you might want to join the study, or why you might not want to join. You may choose to join or not join.

Joining the study is completely up to you. If you agree to join the study, you will be asked to allow your clinic visit to be audio-recorded, and to spend about 15 minutes filling out two different surveys. You can complete the first survey in-person at our office, online, or over the phone. You can complete the second survey online or over the phone. The main risk to you if you join the study is that confidential information about you may be accidentally disclosed.

We don't expect this study to benefit you directly, but it will help us understand how to better support patients as they talk about slow-growing prostate cancer choices. By volunteering, you could help someone else in the future. There is no cost to you. You will be paid with a $15 gift card for being in the study and completing the audio-recording and initial survey, and $5 for completing the second survey. All of this information will be explained and is listed in more detail in this consent. The research team will provide you with a copy of this consent. We can provide it via email or mail.

WHAT IS THE PURPOSE OF THIS STUDY?

This is a research study. We invite you to participate in this research study because you have slow-growing prostate cancer.

The goal of this study is to understand whether and how a decision tool about slow-growing prostate cancer affects care cost discussions and patients' care decisions

WHAT WILL HAPPEN DURING THIS STUDY?

1) You will be asked to review a decision tool given to you by us or your care team. We will ask you if we can audio-record the visit you have with your urologic surgeon. If you agree, the research staff will start an audio-recorder when you enter the exam room or start your virtual visit, then turn off the recorder at the end of the visit. You can still join the study even if you do not want your visit recorded.

2) Right after your appointment, clinic staff will give you a survey to complete in the clinic, or send you a survey by email or phone. This survey should only take about 10 minutes. After you complete the survey, research staff will give you a $15 gift card to thank you for your time.

3) Three months after your appointment, research staff will email you a link to the follow-up survey to complete online. If you do not have an email address or prefer to complete the survey by phone, research staff will read you the survey questions over the phone. This survey should only take about 5 minutes. After you complete the survey, research staff will email or mail you a $5 gift card to thank you for your time.

If at any point in the study you no longer wish to participate, you can stop the recording and/or stop answering survey questions. You can send us an email or call us by phone to let us know you no longer wish to participate. You will not be penalized or lose any benefits.

If you need more time to think about your participation, we will follow up with you with a phone call and/or email in two weeks. We will reach out three times before removing you from the study.

Will you save my research information to use in future research studies?

Your private information will NOT be used for future research studies or shared with other researchers for their studies, even if we remove identifiers.

Audio/Video Recording or Photographs

One aspect of this study involves making audio recordings of you. This is done to learn from the conversations you are having with your surgeon to help people in the future have conversations with their clinicians about cancer care. Only the study team has access to the recordings, which will be destroyed when the study is over. The audio recording is optional and you can still be in the study without being recorded.

I give you permission to make an audio recording of me during this study.

_____ Yes **_____ No**
Initials **Initials**

HOW MANY PEOPLE WILL PARTICIPATE?

Approximately 200 people will take part in this study conducted by investigators at Washington University and Dartmouth College.

HOW LONG WILL I BE IN THIS STUDY?

If you agree to take part in this study, your involvement will last for the length of your clinic visit, and about 15 more minutes for completion of two surveys: one survey immediately after your visit (10 minutes) and another survey three months later (5 minutes).

WHAT ARE THE RISKS OF THIS STUDY?

You may experience one or more of the risks indicated below from being in this study. In addition to these, there may be other unknown risks, or risks that we did not anticipate, associated with being in this study.

One risk of participating in this study is that confidential information about you may be accidentally disclosed. We will use our best efforts to keep the information about you secure. Please see the section in this consent form titled *"How will you keep my information confidential?"* for more information.

WHAT ARE THE BENEFITS OF THIS STUDY?

You will not benefit from being in this study. However, we hope that, in the future, other people might benefit from this study because it may help us understand how to better support patients as they talk about care costs.

WILL IT COST ME ANYTHING TO BE IN THIS STUDY?

You will not have any costs for being in this research study.

WILL I BE PAID FOR PARTICIPATING?

You will be paid for being in this research study. You will receive a $15 gift card after completing the first survey and a $5 gift card after completing the second survey to thank you for your time.

You will need to provide your social security number (SSN) in order for us to pay you. You may choose to participate without being paid if you do not wish to provide your social security number (SSN) for this purpose. You may also need to provide your address if a gift card will be mailed to you. It will take approximately 2 weeks for the gift card to be delivered. If your social security number is obtained for payment purposes only, it will not be retained for research purposes.

WHO IS FUNDING THIS STUDY?

The Robert Wood Johnson Foundation is funding this research study. This means that the Washington University is receiving payments from the Robert Wood Johnson Foundation to support the activities that are required to conduct the study. No one on the research team will receive a direct payment or increase in salary from the Robert Wood Johnson Foundation for conducting this study.

HOW WILL YOU KEEP MY INFORMATION CONFIDENTIAL?

Other people such as those indicated below may become aware of your participation in this study and may inspect and copy records pertaining to this research. Some of these records could contain information that personally identifies you.

- Government representatives (including the Office for Human Research Protections) to complete federal or state responsibilities
- University representatives to complete University responsibilities
- Washington University's Institutional Review Board (a committee that oversees the conduct of research involving human participants) and Human Research Protection Office. The Institutional Review Board has reviewed and approved this study.
- Any report or article that we write will not include information that can directly identify you. The journals that publish these reports or articles require that we share your information that was collected for this study with others to make sure the results of this study are correct and help develop new ideas for research. Your information will be shared in a way that cannot directly identify you.

To help protect your confidentiality, we will use an ID code to identify your surveys and audio-recordings instead of your name. We will destroy the link between the ID code and your name after the study is over. All data will be stored electronically under password protection in a secured server. Study-related computers will be under firewall protection and will maintain automated virus update mechanisms. Timely notification regarding relevant patches will be provided. Hard copies of data collection forms will be stored in locked cabinets in locked areas to which only authorized personnel will have access. In addition, all study staff annually sign a confidentiality statement attesting to their understanding of, and willingness to abide by, written policies on research ethics and confidentiality. Access to the data entry website will be password protected and restricted to personnel trained to use the system.

Our collaborators at Dartmouth College will be involved in the data analysis once the study is finished, but will only have access to de-identified data that cannot be linked to you.

The Siteman Cancer Center at Washington University School of Medicine and Barnes-Jewish Hospital is supported by funding from the National Cancer Institute (NCI). To meet NCI requirements, identifiable information about you relating to your participation in this study (including your social security number) will be stored in a secure database at the Siteman Cancer Center. This database and also your health care records may be reviewed by Siteman Cancer Center personnel. All information will be securely and confidentially maintained.

You have the right to share your information or involvement in this study with anyone at any time. You may also give the research team permission to disclose your information to a third party or any other person not connected with the research.

Are there additional protections for my health information?

Protected Health Information (PHI) is health information that identifies you. PHI is protected by federal law under HIPAA (the Health Insurance Portability and Accountability Act). To take part in this research, you must give the research team permission to use and disclose (share) your PHI for the study as explained in this consent form. The research team will follow state and federal laws and may share

your health information with the agencies and people listed under the previous section titled, “How will you keep my information confidential?”

Once your health information is shared with someone outside of the research team, it may no longer be protected by HIPAA.

The research team will only use and share your information as talked about in this form or as permitted or required by law. When possible, the research team will make sure information cannot be linked to you (de-identified). Once information is de-identified, it may be used and shared for other purposes not discussed in this consent form. If you have questions or concerns about your privacy and the use of your PHI, please contact the University’s Privacy Officer at [phone number].

Although you will not be allowed to see the study information, you may be given access to your health care records by contacting your health care provider.

If you decide not to sign this form, it will not affect

your treatment or the care given by your health provider.
your insurance payment or enrollment in any health plans.
any benefits to which you are entitled.
However, it will not be possible for you to take part in the study.

If you sign this form:

- You authorize the use of your PHI for this research
- This authorization does not expire.

You may later change your mind and not let the research team use or share your information (you may revoke your authorization).

- To revoke your authorization, complete the withdrawal letter, found in the Participant section of the Human Research Protection Office website at https://hrpo.wustl.edu/participants/withdrawing-from-a-study/ or you may request that the investigator send you a copy of the letter.
    - **If you revoke your authorization:**
        - ♣ The research team may only use and share information already collected for the study.
        - ♣ Your information may still be used and shared as necessary to maintain the integrity of the research, for example, to account for a participant’s withdrawal from the research study or for safety reasons.
        - ♣ You will not be allowed to continue to participate in the study.

IS BEING IN THIS STUDY VOLUNTARY?

Taking part in this research study is completely voluntary. You may choose not to take part at all. If you decide to be in this study, you may stop participating at any time. Any data that was collected as part of your participation in the study will remain as part of the study records and cannot be removed.

If you decide not to be in this study, or if you stop participating at any time, you won’t be penalized or lose any benefits.

<u>What if I decide to withdraw from the study?</u>

You may withdraw by telling the study team you are no longer interested in participating in the study.

If at any point in the study you no longer wish to participate, you can stop the audio-recording and/or stop answering survey questions. You can send us an email or give us a phone call to let us know you no longer wish to participate. You will not be penalized or lose any benefits.

If you do not tell us you no longer wish to participate in the study or need more time to think about your participation, we will follow-up with you with a phone call and/or email in two weeks. We will reach out three times before removing you from the study.

<u>Will I receive new information about the study while participating?</u>

If we obtain any new information during this study that might affect your willingness to continue participating in the study, we'll promptly provide you with that information.

<u>WHAT IF I HAVE QUESTIONS?</u>

We encourage you to ask questions. If you have any questions about the research study itself, please contact: Katie Parrish, MPH at [phone number]. If you feel that you have been harmed in any way by your participation in this study, please contact Mary Politi, PhD at [phone number].

If you have questions, concerns, or complaints about your rights as a research participant please contact the Human Research Protection Office at [phone number], or email [email address]. General information about being a research participant can be found on the Human Research Protection Office web site, http://hrpo.wustl.edu. To offer input about your experiences as a research participant or to speak to someone other than the research staff, call the Human Research Protection Office at the number above.

<u>CAN WE CONTACT YOU BY EMAIL?</u>

We would like to contact you by email for the purposes listed below. Some of these emails may contain health information that identifies you.

- Links to surveys
- Study information, including a copy of this consent form
- Reminder emails about completing your post-survey, if you are completing them at home

Only the research team will have access to your email communications. We will only communicate by email to send you the information listed above. If you have any questions or need to contact us for an urgent or emergent situation, please contact the research team member identified at the top of this document.

You should be aware that there are risks associated with sending your health information via email.

- There is always a risk that the message could be intercepted or sent to the wrong email address. To avoid sending messages to the wrong email address, the first email we send you will be a test message to ensure we have the correct email address.

- When using any computer you should be careful to protect your username and password. Make sure you log-out before getting up from the computer.
- If you share a home computer with other family members, and do not want them to know you are participating in this study make sure you provide an email address that only you can access.
- Your employer will have access to any email communications sent or received on any electronic devices used for work or through a work server.

Do you agree to allow us to send your health information via email?

_____ Yes _____ No
Initials Initials

This consent form is not a contract. It is a written explanation of what will happen during the study if you decide to participate. You are not waiving any legal rights by agreeing to participate in this study.

Your signature indicates that this research study has been explained to you, that your questions have been answered, and that you agree to take part in this study. You will receive a signed copy of this form.

Do not sign this form if today's date is after EXPIRATION DATE: N/A.

_______________________________________ _______________________________
(Signature of Participant) (Date)

____
(Participant's name – printed)

Statement of Person Who Obtained Consent

The information in this document has been discussed with the participant or, where appropriate, with the participant's legally authorized representative. The participant has indicated that they understand the risks, benefits, and procedures involved with participation in this research study.

(Signature of Person who Obtained Consent) (Date)

(Name of Person who Obtained Consent - printed)
